# Dietary Arginine Supplementation Mitigates Heat Stress-Induced Testicular Dysfunction Through Arginine-Related Metabolic Remodeling and Autophagy-Associated Stress Adaptation in a Rongchang Boar Model

**DOI:** 10.3390/ani16172682

**Published:** 2026-08-27

**Authors:** Xiao Lin, Kun Wu, Jingchang Ren, Yong Zhuo, Bin Feng, Lianqiang Che, Zhengfeng Fang, Shengyu Xu, Lun Hua, Guangmang Liu, Xiaojun Jiang, Jian Li, De Wu, Yan Lin

**Affiliations:** 1 Animal Nutrition Institute, Sichuan Agricultural University, Chengdu 611130, China; linxiao@stu.sicau.edu.cn (X.L.); 18030726343@163.com (K.W.); 15983100307@163.com (J.R.); zhuoyong@sicau.edu.cn (Y.Z.); fengbin@sicau.edu.cn (B.F.); che.lianqiang@sicau.edu.cn (L.C.); zfang@sicau.edu.cn (Z.F.); shengyuxu@sicau.edu.cn (S.X.); hualun@sicau.edu.cn (L.H.); liugm@sicau.edu.cn (G.L.); lijian522@hotmail.com (J.L.); pig2pig@sina.com (D.W.); 2 College of Food Science, Sichuan Agricultural University, Ya’an 625014, China; 3 Sichuan DaBeiNong Agriculture and Animal Husbandry Technology Co., Ltd., Chengdu 611430, China; jxj4@21cn.com

**Keywords:** heat stress, dietary arginine, testicular dysfunction, arginine metabolism, autophagy, Rongchang boar

## Abstract

Heat stress compromises male reproductive function in livestock and other mammals. Arginine is a conditionally essential amino acid involved in metabolic regulation; however, its protective effects on the male reproductive system under heat stress remain unclear. Using a heat-stressed Rongchang boar model, this study aimed to explore the possible mechanism of dietary arginine supplementation on sperm quality and testicular function. Twenty-four Rongchang boars were assigned to three treatments: thermoneutral control (CON), heat-stressed control (HS), or heat-stressed treatment supplemented with 0.8% dietary arginine (HA). Results showed that heat stress reduced feed intake, body weight, sperm viability, sperm concentration, and sperm motion parameters (*p* < 0.05), accompanied by testicular histological damage. However, arginine supplementation improved sperm viability and straight-line velocity (*p* < 0.05), with partial preservation of testicular morphology. Furthermore, single-cell RNA sequencing revealed heat stress-associated changes in germ-cell composition and enrichment of autophagy-related pathways. Transmission electron microscopy and qRT-PCR showed endoplasmic reticulum swelling, autophagic vesicle accumulation, and increased expression of *GABARAPL1*, *ULK1*, *BECN1*, and *CALM3* in heat-stressed testes (*p* < 0.05); these alterations were attenuated by arginine supplementation, as indicated by reduced *ULK1* and *BECN1* expression (*p* < 0.05). Metabolomic and targeted analyses indicated improved circulating arginine availability and reshaped testicular arginine-related amino acid/polyamine metabolism after arginine supplementation (*p* < 0.05). Overall, dietary arginine supplementation alleviated heat stress-induced testicular dysfunction, which was accompanied by alterations in arginine-related metabolic regulation and autophagy-associated cellular stress responses.

## Simple Summary

Heat stress is an important environmental factor that compromises male reproductive function, particularly sperm quality and testicular health. Arginine, a conditionally essential amino acid, participates in metabolic regulation and stress adaptation; however, its protective role in the male reproductive system under heat-stress conditions remains unclear. This study used a heat-stressed Rongchang boar model to evaluate whether dietary arginine supplementation could alleviate reproductive impairment. Heat stress impaired epididymal sperm quality and seminiferous tubule integrity. Arginine supplementation enhanced sperm viability and straight-line velocity, accompanied by partial preservation of testicular morphology. Metabolomic analysis indicated disrupted amino acid and arginine-related metabolism in heat-stressed testes. Arginine supplementation, in turn, increased circulating arginine availability while reshaping testicular arginine-related amino acid/polyamine metabolism. Evidence from single-cell transcriptomics, ultrastructural observation, and gene expression profiling further suggested that arginine supplementation attenuated autophagy-associated cellular responses induced by heat stress. These findings suggest that dietary arginine may protect sperm quality and testicular function under heat-stress conditions by coordinating arginine-related metabolic regulation with autophagy-associated stress adaptation.

## 1. Introduction

Heat stress is a major environmental challenge that impairs animal health, productivity, and reproductive performance, thereby leading to substantial economic losses in livestock production [1,2,3]. Among the physiological systems affected by heat stress, male reproductive function is particularly vulnerable because spermatogenesis and testicular homeostasis depend on a tightly regulated thermal environment [4,5]. In addition, testicular heat exposure has been reported to impair Leydig cell steroidogenic function and reduce serum and testicular testosterone concentrations [6]. When ambient temperature exceeds the thermoneutral zone, animals initiate a series of behavioral, physiological, and metabolic responses to maintain heat balance. Nevertheless, if heat exposure is prolonged or severe, these adaptive responses may become insufficient, resulting in suppressed feed intake, reduced growth performance, and compromised male reproductive capacity [1,2,3].

The testis is highly vulnerable to thermal injury [4,5]. This vulnerability is closely related to the fact that germ-cell development, seminiferous epithelial organization, and epididymal sperm maturation all require a stable testicular microenvironment. Accordingly, exposure to elevated temperature has been shown to impair spermatogenesis, reduce sperm concentration and motility, and disrupt seminiferous tubule integrity [7,8,9,10,11]. In addition to these functional and structural impairments, heat stress may also alter germ-cell composition and damage the overall integrity of the testes [8,10,11]. Therefore, nutritional strategies that preserve sperm quality and testicular function under heat-stress conditions are important for maintaining male reproductive performance.

Amino acid metabolism is closely linked to male reproductive function. In this context, amino acids not only serve as substrates for protein synthesis but also act as metabolic regulators of cell proliferation, differentiation, redox balance, and stress responses [12,13,14]. Among these amino acids, arginine is considered conditionally essential during physiological stress, when endogenous synthesis may be insufficient to meet increased metabolic demands [15]. Moreover, arginine serves as a precursor for nitric oxide, ornithine, and polyamines through nitric oxide synthase- and arginase-related pathways [14]. These downstream metabolites are involved in vascular regulation, antioxidant defense, cellular growth, and reproductive function [16,17]. Therefore, dietary arginine supplementation may represent a potential nutritional strategy for mitigating heat stress-induced testicular impairment. Beyond its metabolic functions, arginine availability may influence cellular stress adaptation through nutrient-sensing and redox-regulatory pathways [18]. Emerging evidence suggests that amino acid metabolism is closely connected with autophagy regulation, as nutrient availability can affect autophagy-associated cellular responses under stress conditions [19]. Therefore, arginine metabolism may represent a potential link between nutritional intervention and autophagy-associated adaptation during heat stress.

Among cellular stress adaptation pathways, autophagy is a conserved lysosome-dependent degradation process that plays a critical role in maintaining cellular homeostasis under metabolic and environmental stress conditions [20,21,22,23,24]. Under basal or moderate stress conditions, this process removes damaged proteins and organelles, thereby promoting cell survival [20,21]. However, when autophagy becomes excessive or dysregulated, it may aggravate cellular injury and contribute to tissue dysfunction [21,22,23]. Consistent with this dual role, heat stress has been reported to alter autophagy-related signaling in testicular cells, which may interfere with spermatogenesis and germ-cell survival [5,8,15]. Nevertheless, whether modulation of autophagy-associated responses contributes to the protective effects of dietary arginine against heat stress-induced testicular dysfunction remains insufficiently understood.

However, whether dietary arginine supplementation can protect the testes against heat stress-induced dysfunction through coordinated regulation of arginine-related metabolism and autophagy-associated stress responses remains unclear. Based on these considerations, the present study used a heat-stressed Rongchang boar model to evaluate the effects of dietary arginine supplementation on sperm quality and testicular histomorphology. We hypothesized that dietary arginine supplementation could alleviate heat stress-induced testicular dysfunction by improving arginine-related metabolic homeostasis and modulating autophagy-associated stress responses. Single-cell RNA sequencing, untargeted metabolomics, transmission electron microscopy, and qRT-PCR were further integrated to investigate potential mechanisms involving testicular cellular composition, arginine-related amino acid/polyamine metabolism, and autophagy-associated responses.

## 2. Materials and Methods

### 2.1. Animal Ethics Statement

All animal care and experimental procedures were approved by the Institutional Animal Care and Use Committee of Sichuan Agricultural University (approval no. SI20230168). The study was conducted in accordance with the university guidelines for the care and use of laboratory animals.

### 2.2. Animals and Experimental Design

Twenty-four 43-day-old Rongchang boars with similar initial body weight (BW; 5.87 ± 0.16 kg, mean ± SEM) were used in this study. After a 5-d acclimation period under thermoneutral conditions (25 ± 1 °C; 50 ± 1% relative humidity), the animals underwent a 72-d experiment comprising two periods: a 63-d thermoneutral period (P1) and a 9-d heat-stress challenge (P2). The boars were blocked by initial BW, then randomly assigned within blocks to three treatments. Each treatment included eight replicates, with one boar per replicate. The treatments were as follows: CON, basal diet under thermoneutral conditions; HS, basal diet under cyclic heat stress; HA, 0.8% arginine-supplemented diet under cyclic heat stress. The supplementation level of 0.8% L-arginine was selected based on previous studies demonstrating the beneficial effects of dietary arginine supplementation on reproductive function and testicular development in pigs [16].

The basal diet (Table 1) was formulated according to the nutrient requirements for Chinese local pig breeds. Considering that Rongchang pigs are Chinese indigenous pigs with distinct growth characteristics and nutrient utilization patterns compared with commercial European breeds, the dietary formulations were designed based on the nutritional requirements of Rongchang boars. The crude protein level was maintained consistently between growth stages to minimize dietary protein-related variation and ensure experimental comparability. The diet was calculated to provide 3.24 Mcal/kg metabolizable energy (ME) and 17.91% crude protein (CP). All boars were housed individually in crates under a 15:9 h light–dark cycle throughout the experiment. Diets were provided in four equal meals daily at 08:00, 12:00, 16:00, and 20:00. Feed offered and refusals were recorded daily to calculate individual daily feed intake. Fresh water was available ad libitum.

During P1, all boars were maintained under thermoneutral conditions and received their assigned diets to obtain baseline thermoregulatory and growth-related data. During P2, CON boars remained under thermoneutral conditions with the basal diet. In contrast, HS and HA boars were exposed to cyclic heat stress while receiving the basal diet or the arginine-supplemented diet, respectively. For the heat-stressed treatments, mean ambient temperatures were 38.7 °C during the daytime period (07:00–19:00) and 33.3 °C during the nighttime period (19:00–07:00). The environmental temperature was controlled using air-conditioning and heating equipment.

During P2, rectal temperature and respiratory rate were recorded every 3 d. Rectal temperature was measured by inserting a mercury thermometer into the rectum for 5 min. Respiratory rate was assessed by counting flank movements for 1 min. Ambient temperature and relative humidity were continuously monitored using a data logger (COS-03; Renke, Shandong, China). The temperature–humidity index (THI) was calculated as follows:

THI = (1.8 × AT + 32) − (0.55 − 0.0055 × RH) × (1.8 × AT − 26.8)

where AT represents ambient temperature (°C), and RH represents relative humidity (%).

### 2.3. Sample Collection and Epididymal Sperm Quality Assessment

After anesthesia was induced by intramuscular injection of Zoletil 50 (tiletamine hydrochloride and zolazepam hydrochloride for injection; Virbac, Carros, France) at 0.05 mL/kg body weight, the boars were humanely euthanized by exsanguination. The epididymides were collected immediately and transported to the laboratory. Both cauda epididymides were separated, minced, and processed together for sperm collection. The minced tissues were transferred into tubes containing 10 mL of pre-warmed semen extender (China Animal Husbandry Group, Beijing, China). After incubation in a 37 °C water bath for 15 min, the sperm suspension was collected and diluted five-fold with the same extender. Epididymal sperm quality was then assessed using a computer-assisted sperm analysis (CASA) system (Minitube AndroVision, Tiefenbach, Germany).

### 2.4. Histological Staining and Analysis

Testicular tissue samples were fixed in 4% paraformaldehyde, dehydrated through a graded ethanol series, embedded in paraffin, and sectioned at 5 μm. The sections were mounted on glass slides and stained with hematoxylin and eosin (H&E) according to standard histological procedures. Testicular histomorphology was examined under a light microscope. The following histomorphometric parameters were quantified using NDP.view2 software (version 2.9.29): seminiferous tubule diameter, seminiferous epithelial height, and vacuolated area within seminiferous tubules.

### 2.5. Single-Cell Transcriptomic Analysis

Fresh testicular tissue samples were enzymatically dissociated to obtain viable single-cell suspensions for single-cell RNA sequencing (scRNA-seq). Library preparation and sequencing were performed by LC-Bio Technology Co., Ltd. (Hangzhou, China). Cells were encapsulated in gel beads-in-emulsion (GEMs), and mRNA molecules were captured using barcoded gel beads. The captured mRNA was reverse-transcribed into cDNA, followed by amplification and library construction. The scRNA-seq libraries were sequenced on an Illumina NextSeq 500 platform (Illumina, San Diego, CA, USA).

Raw scRNA-seq reads were processed using Cell Ranger. Reads were aligned to the *Sus scrofa* reference genome, and gene-by-cell count matrices were generated after barcode processing and unique molecular identifier (UMI) counting. Downstream analyses were performed using the Seurat package in R. Low-quality cells were removed before normalization, dimensionality reduction, clustering, and visualization of cell populations. Cell identities were annotated by comparing cluster-specific marker genes with reference information from the Cell Taxonomy database (https://ngdc.cncb.ac.cn/celltaxonomy/, accessed on 14 March 2026). Differentially expressed genes (DEGs) between treatments were identified using the bimod test with *p* < 0.01 and |log2 fold change| ≥ 0.26. Functional enrichment analysis was performed based on the identified DEGs. Annotated cell populations were further subsetted and reclustered to identify cell-type-specific DEGs, followed by Kyoto Encyclopedia of Genes and Genomes (KEGG) pathway enrichment analysis.

### 2.6. Metabolomic Analysis

Testicular tissue samples were thawed at 4 °C for untargeted metabolomic profiling. Approximately 50 mg of each sample was homogenized with 1 mL of pre-chilled methanol/acetonitrile/water extraction solvent (2:2:1, *v*/*v*/*v*). The homogenate was vortexed, sonicated in an ice-water bath for 30 min, and incubated at −20 °C for 10 min to precipitate proteins. After centrifugation at 14,000× *g* for 20 min at 4 °C, the supernatant was transferred to a clean tube and evaporated to dryness under vacuum. The dried extract was reconstituted in 100 μL of acetonitrile/water (1:1, *v*/*v*), vortexed, and centrifuged at 14,000× *g* for 15 min at 4 °C. The clear supernatant was then transferred to autosampler vials for LC–MS analysis.

Raw LC–MS data were converted to mzXML format using ProteoWizard (version 3.0.25011). Peak detection, retention-time correction, peak alignment, and peak integration were performed using XCMS software (version 4.6.4). Metabolite annotation and preprocessing were conducted based on the XCMS-generated feature table. Features with missing values in more than 50% of samples were removed. The remaining missing values were imputed using the k-nearest neighbor (KNN) method. To improve data reliability, features with a relative standard deviation (RSD) greater than 50% in quality control samples were excluded. Analytical stability was then assessed before downstream statistical analysis. The normalized metabolomic dataset was also used to extract the relative abundances of selected arginine-related metabolites, including testicular L-citrulline and ornithine.

### 2.7. Determination of L-Arginine Concentration and Content

Based on the untargeted metabolomic findings, L-arginine levels in serum and testicular tissues were further quantified. Serum L-arginine concentration and testicular L-arginine content were determined using an amino acid auto-analyzer (L-8800; Hitachi, Tokyo, Japan). For testicular analysis, tissue samples were hydrolyzed in 6 N hydrochloric acid at 110 °C for 24 h under a nitrogen atmosphere. The resulting hydrolysates were then subjected to ion-exchange chromatographic separation before instrumental detection. Serum samples were pretreated according to a previously described method with minor modifications [21]. Blank solutions containing 0.1 M HCl and 160 μM norleucine were prepared together with external amino acid standards, including acidic, neutral, and basic amino acid standards (Sigma-Aldrich, St. Louis, MO, USA). Serum L-arginine concentration was expressed as μmol/L, whereas testicular L-arginine content was expressed as mg/kg tissue.

### 2.8. Quantitative Real-Time PCR

Total RNA was extracted from testicular tissue samples using a commercial RNA isolation kit (UE-MN-MS-RNA-250; UElandy, Suzhou, China). RNA concentration and purity were assessed using a NanoDrop 2000c spectrophotometer (Thermo Fisher Scientific, Waltham, MA, USA). RNA integrity was evaluated by agarose gel electrophoresis. First-strand cDNA was synthesized from 1 µg of total RNA using a reverse transcription kit (AT341; TransGen Biotech, Beijing, China). Quantitative real-time PCR (qRT-PCR) was performed in a 10-µL reaction system using SYBR Green qPCR mix (AQ602-02-V2; TransGen Biotech, Beijing, China). Each sample was analyzed with three technical replicates. Relative mRNA expression levels were calculated using the 2^−ΔΔCt^ method and normalized to β-actin. Genes associated with autophagy regulation and cellular stress responses, including ULK1, BECN1, GABARAPL1, and CALM3, were selected based on their reported biological functions. Primer sequences are listed in Table 2.

### 2.9. Transmission Electron Microscopy

Fresh testicular tissue samples were collected within 1–3 min after dissection and trimmed into approximately 1 mm^3^ pieces to minimize tissue distortion. The specimens were immediately immersed in electron microscopy fixative and stored at 4 °C for transportation. After primary fixation, the samples were washed three times with 0.1 M phosphate buffer (PB; pH 7.4) for 10 min each. They were then post-fixed with 1% osmium tetroxide in 0.1 M PB for 2 h at room temperature in the dark. The tissues were washed again with 0.1 M PB and dehydrated through a graded ethanol series of 30%, 50%, 70%, 80%, 95%, and 100%. Each dehydration step lasted 10 min, and the 100% ethanol step was repeated twice. The samples were then treated twice with 100% acetone for 10 min.

For resin infiltration, the samples were sequentially incubated in acetone/812 resin mixtures at ratios of 1:1 for 2–4 h and 1:2 overnight at 37 °C. The specimens were then transferred into pure 812 resin for 5–8 h at 37 °C. After embedding in fresh 812 resin, the samples were polymerized at 60 °C for 48 h to obtain resin blocks. Ultrathin sections of 70 nm were prepared and mounted on Formvar-coated 200-mesh copper grids for transmission electron microscopy (TEM) analysis.

### 2.10. Statistical Analysis

Statistical analyses were performed using SPSS software version 19.0. Data are presented as the mean ± standard error of the mean (SEM). Normality and homogeneity of variance were assessed using the Shapiro–Wilk test and Levene’s test, respectively. Endpoint measurements were analyzed by one-way analysis of variance (ANOVA), followed by the least significant difference (LSD) multiple-comparison test. Repeated measurements, including rectal temperature and respiratory rate, were analyzed using repeated-measures ANOVA. Statistical significance was set at *p* < 0.05.

## 3. Results

### 3.1. Dietary Arginine Supplementation Modulates Growth Performance and Thermoregulatory Responses in Heat-Stressed Rongchang Boars

**Figure 1:**
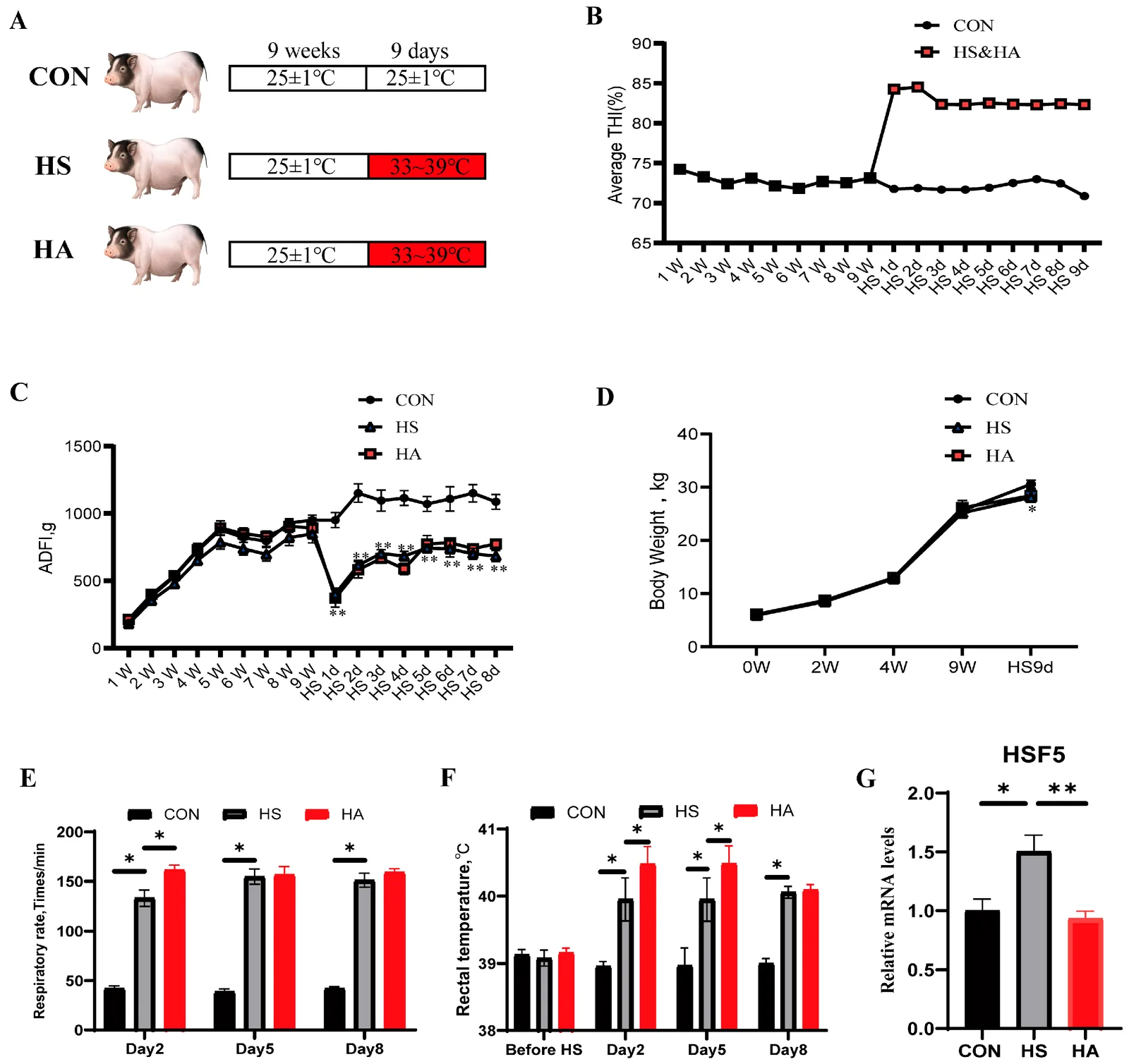
Experimental design, growth performance, thermoregulatory responses, and *HSF5* expression in Rongchang boars exposed to heat stress. (**A**) Schematic illustration of the experimental design. (**B**) Temperature–humidity index (THI) during the experimental period; HS and HA were exposed to the same cyclic heat-stress conditions during P2. (**C**) Average daily feed intake (ADFI). (**D**) Body weight. (**E**) Respiratory rate. (**F**) Rectal temperature. (**G**) Relative *HSF5* mRNA expression. CON, thermoneutral control group fed the basal diet; HS, heat-stressed group fed the basal diet; HA, heat-stressed group fed the diet supplemented with 0.8% arginine. Data are presented as mean ± SEM (*n* = 8 per treatment). Asterisks indicate significant differences for the indicated comparisons: * *p* < 0.05; ** *p* < 0.01.

The physiological responses of Rongchang boars to heat stress and dietary arginine supplementation are shown in Figure 1. During P2, the temperature–humidity index (THI) increased rapidly after heat-stress exposure and remained higher in the HS and HA groups than in the CON group, indicating that the cyclic heat-stress challenge was effectively established (Figure 1B). Accordingly, average daily feed intake was significantly reduced in both heat-stressed groups compared with the CON group (*p* < 0.05; Figure 1C). At the end of P2, body weight was also lower in the HS and HA groups than in the CON group (*p* < 0.05; Figure 1D). Consistent with these growth-related responses, respiratory rate and rectal temperature were markedly increased under heat-stress conditions (*p* < 0.05; Figure 1E,F). Notably, compared with the HS group, the HA group showed a higher respiratory rate on day 2 and higher rectal temperatures on days 2 and 5 (*p* < 0.05; Figure 1E,F), indicating that arginine supplementation did not prevent the thermoregulatory response to heat exposure. At the molecular level, relative HSF5 mRNA expression in the testes was significantly upregulated in the HS group compared with the CON group (*p* < 0.05), whereas dietary arginine supplementation reduced HSF5 expression under heat-stress conditions (*p* < 0.05; Figure 1G). These findings demonstrate that cyclic heat stress induced pronounced thermoregulatory and growth-related responses in Rongchang boars, whereas dietary arginine supplementation partially modulated heat-responsive gene expression in the testes.

### 3.2. Dietary Arginine Supplementation Improves Epididymal Sperm Quality and Partially Preserves Testicular Histomorphology Under Heat Stress

**Figure 2:**
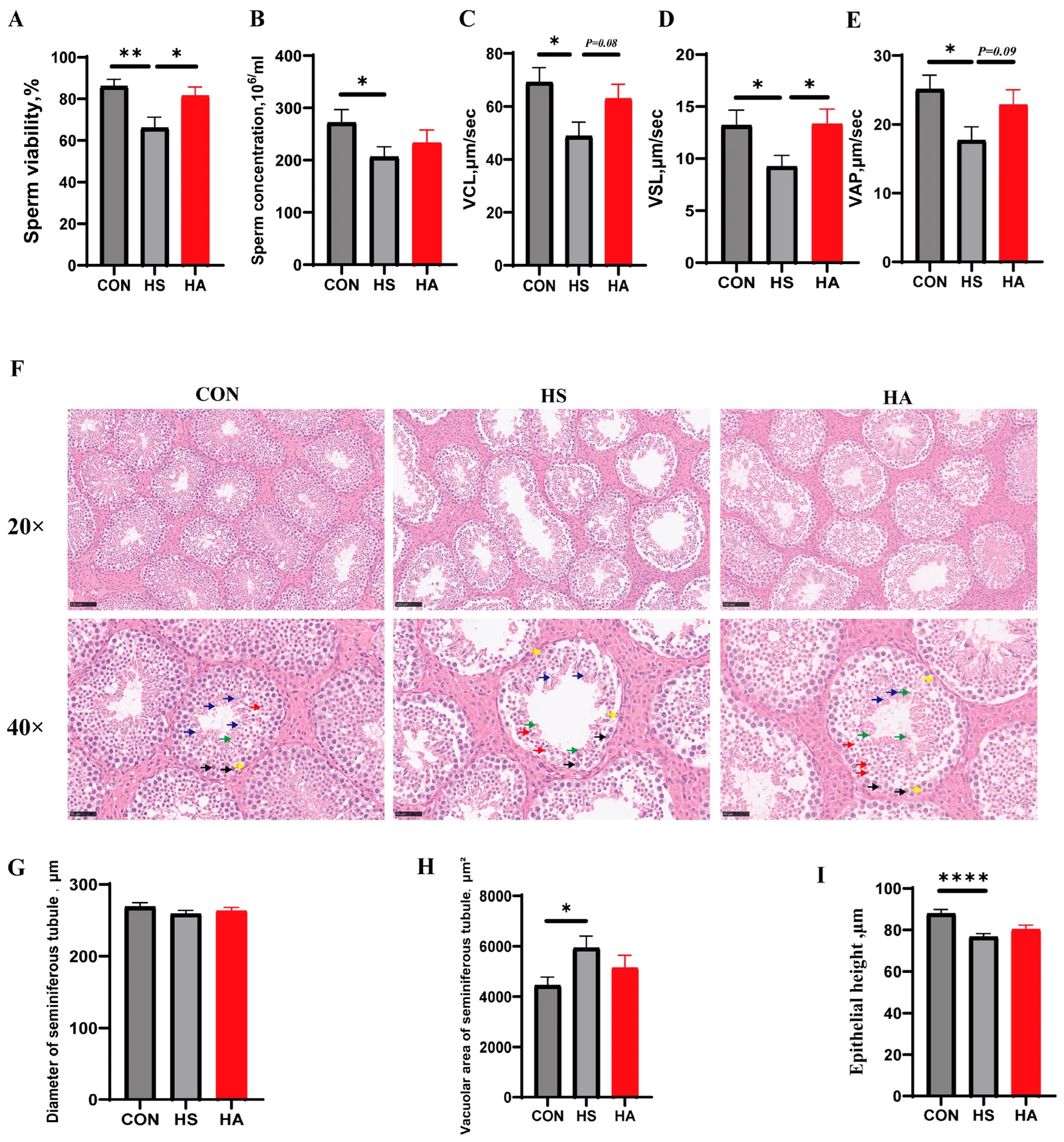
Effects of dietary arginine supplementation on epididymal sperm quality and testicular histomorphology in heat-stressed Rongchang boars. (**A**) Sperm viability. (**B**) Sperm concentration. (**C**) Curvilinear velocity (VCL). (**D**) Straight-line velocity (VSL). (**E**) Average path velocity (VAP). (**F**) Representative hematoxylin and eosin (H&E)-stained testicular sections. (**G**) Seminiferous tubule diameter. (**H**) Vacuolated area within seminiferous tubules. (**I**) Seminiferous epithelial height. CON, thermoneutral control group fed the basal diet; HS, heat-stressed group fed the basal diet; HA, heat-stressed group fed the diet supplemented with 0.8% arginine. Data are presented as mean ± SEM (*n* = 8 per treatment). Asterisks indicate significant differences for the indicated comparisons: * *p* < 0.05; ** *p* < 0.01; **** *p* < 0.0001. Exact *p* values are shown for selected non-significant comparisons.

Epididymal sperm quality and testicular histomorphology were evaluated to characterize heat stress-induced reproductive impairment (Figure 2). Compared with the CON group, the HS group showed significantly lower sperm viability and sperm concentration (*p* < 0.05; Figure 2A,B). In parallel, sperm motion parameters, including curvilinear velocity (VCL), straight-line velocity (VSL), and average path velocity (VAP), were also reduced after heat-stress exposure (*p* < 0.05; Figure 2C–E). However, dietary arginine supplementation significantly increased sperm viability and VSL compared with the HS group (*p* < 0.05; Figure 2A,D). In addition, VCL and VAP were numerically increased in the HA group, although these differences did not reach statistical significance (0.05 < *p* < 0.10; Figure 2C,E).

To further assess testicular structural changes, testicular histomorphology was examined using hematoxylin and eosin (H&E) staining (Figure 2F). Seminiferous tubules in the CON group showed relatively intact architecture and well-organized seminiferous epithelium. In contrast, the HS group exhibited evident histological alterations, including epithelial disruption, enlarged luminal spaces, and increased vacuolated areas. Consistent with these observations, quantitative histomorphometric analysis showed that the HS group had a larger vacuolated area and lower seminiferous epithelial height than the CON group (*p* < 0.05; Figure 2H,I), whereas no significant difference in seminiferous tubule diameter was observed among the three groups (*p* > 0.05; Figure 2G). Representative H&E sections suggested partial preservation of testicular morphology in the HA group. Nevertheless, the quantified vacuolated area and seminiferous epithelial height did not differ significantly between the HS and HA groups (*p* > 0.05; Figure 2F,H,I). The results showed that dietary arginine supplementation improved selected epididymal sperm quality parameters under heat-stress conditions, while its effects on testicular histomorphology were limited to partial morphological preservation.

### 3.3. Single-Cell Transcriptomic Analysis Reveals Altered Testicular Cell Composition and Autophagy-Related Pathways in Response to Heat Stress and Arginine Supplementation

**Figure 3:**
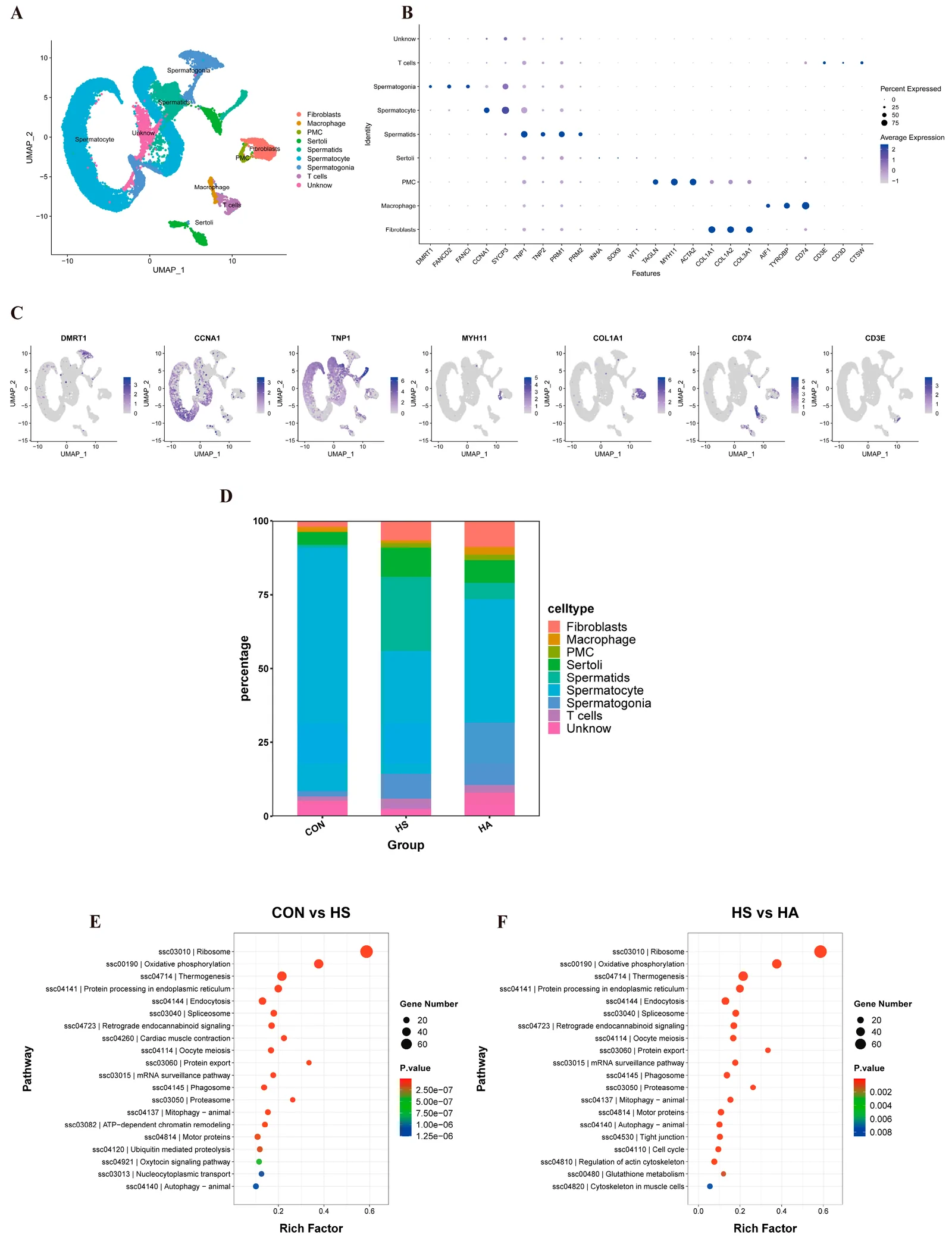
Single-cell transcriptomic profiling of testicular cell populations in response to heat stress and dietary arginine supplementation. (**A**) Uniform manifold approximation and projection (UMAP) visualization of annotated testicular cell populations. (**B**) Dot plot showing the expression patterns of representative marker genes across annotated cell types. Dot size represents the percentage of cells expressing each marker gene, and color intensity indicates the average scaled expression level. (**C**) Feature plots showing the expression of selected cell-type-specific marker genes, including *DMRT1*, *CCNA1*, *TNP1*, *MYH11*, *COL1A1*, *CD74*, and *CD3E*. (**D**) Relative distribution of annotated testicular cell populations among the CON, HS, and HA groups. (**E**,**F**) Kyoto Encyclopedia of Genes and Genomes (KEGG) pathway enrichment analysis of differentially expressed genes (DEGs) in the CON vs. HS comparison (**E**) and the HS vs. HA comparison (**F**). Dot size represents gene number, and color indicates the *p* value. CON, thermoneutral control group fed the basal diet; HS, heat-stressed group fed the basal diet; HA, heat-stressed group fed the diet supplemented with 0.8% arginine; PMC, peritubular myoid cells; Unknown, unannotated cell clusters.

To characterize testicular cellular heterogeneity in response to heat stress and dietary arginine supplementation, scRNA-seq analysis was performed using testicular cell suspensions. Based on cluster-specific marker gene expression, major testicular cell populations were annotated, including spermatogonia, spermatocytes, spermatids, Sertoli cells, peritubular myoid cells, fibroblasts, macrophages, and T cells (Figure 3A). Consistently, dot plot analysis showed distinct expression patterns of representative marker genes across these annotated cell populations (Figure 3B). Specifically, spermatogonia were characterized by high expression of *DMRT1*, *FANCD2*, and *FANCI*, whereas spermatocytes highly expressed *CCNA1* and *SYCP3*. Spermatids were identified by the expression of *TNP1*, *TNP2*, *PRM1*, and *PRM2*. Sertoli cells showed high expression of *INHA*, *SOX9*, *WT1*, and *FATE1*. In addition, peritubular myoid cells, fibroblasts, macrophages, and T cells were annotated based on the expression of *TAGLN*/*MYH11*/*ACTA2*, *COL1A1*/*COL1A2*/*COL3A1*, *AIF1*/*TYROBP*/*CD74*, and *CD3E*/*CD3D*/*CTSW*, respectively. Feature plots of selected marker genes further supported the annotation of major testicular cell types (Figure 3C). Cell clusters that could not be confidently assigned to known testicular cell populations were retained as unannotated clusters.

Cell proportion analysis further showed that heat stress altered testicular cellular composition (Figure 3D). Compared with the CON group, the relative proportion of spermatocytes was reduced in the HS and HA groups, whereas the abundance of spermatogonia was increased, particularly in the HA group. To explore the potential functional relevance of these cellular changes, KEGG pathway enrichment analysis was performed using differentially expressed genes. In the CON vs. HS comparison, enriched pathways included autophagy-related pathways, such as mitophagy (Figure 3E). Similarly, pathways associated with autophagy-related regulation were also enriched in the HS vs. HA comparison (Figure 3F). Single-cell RNA sequencing analysis revealed that heat stress and dietary arginine supplementation were associated with alterations in testicular cellular composition and autophagy-associated responses.

### 3.4. Dietary Arginine Supplementation Attenuates Heat Stress-Induced Autophagy-Related Ultrastructural and Gene Expression Changes

**Figure 4:**
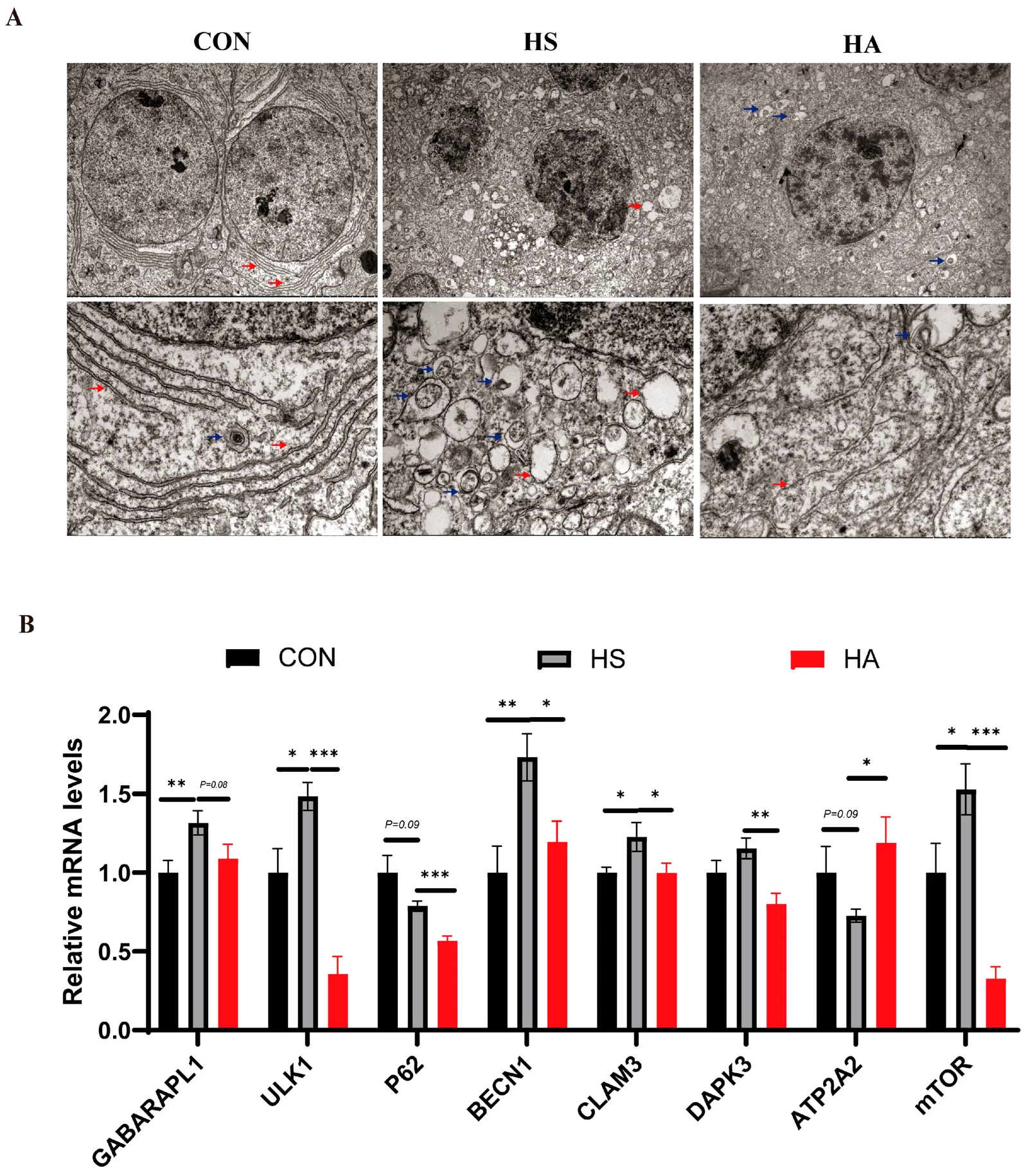
Effects of dietary arginine supplementation on heat stress-induced autophagy-related ultrastructural and gene expression changes in the testes of Rongchang boars. (**A**) Representative transmission electron microscopy (TEM) images of testicular cells. Red arrows indicate endoplasmic reticulum (ER) structures, and blue arrows indicate autophagic vesicles. (**B**) Relative mRNA expression levels of autophagy-related genes and ER-associated genes in the testes. CON, thermoneutral control group fed the basal diet; HS, heat-stressed group fed the basal diet; HA, heat-stressed group fed the diet supplemented with 0.8% arginine. Data are presented as mean ± SEM. Statistical analyses were performed using one-way ANOVA followed by LSD multiple-comparison tests. Asterisks indicate significant differences for the indicated comparisons: * *p* < 0.05; ** *p* < 0.01; *** *p* < 0.001. Exact *p* values are shown for selected non-significant comparisons.

To further examine the autophagy-related alterations suggested by scRNA-seq analysis, transmission electron microscopy (TEM) and qRT-PCR were performed. TEM observations showed that testicular cells in the CON group had relatively intact endoplasmic reticulum (ER) morphology and well-organized ultrastructure, with only a few autophagic vesicles (Figure 4A). In contrast, the HS group exhibited pronounced ER swelling, ultrastructural disorganization, and increased autophagic vesicle accumulation. Although ER swelling and structural disorganization were still observed in the HA group, autophagic vesicle accumulation appeared to be reduced compared with the HS group (Figure 4A).

Consistent with these ultrastructural findings, qRT-PCR was used to examine genes associated with autophagy regulation and ER-related cellular stress. The relative mRNA expression levels of *GABARAPL1*, *ULK1*, *BECN1*, and *CALM3* were significantly higher in the HS group than in the CON group (*p* < 0.05; Figure 4B). These changes indicate enhanced autophagy-associated and ER-related transcriptional responses under heat-stress conditions. Compared with the HS group, dietary arginine supplementation significantly downregulated *ULK1* and *BECN1* expression (*p* < 0.05; Figure 4B). In addition, *GABARAPL1* and *ATP2A2* expression showed numerical decreases in the HA group, although these differences did not reach statistical significance (0.05 < *p* < 0.10; Figure 4B). Collectively, these findings suggest that dietary arginine supplementation may attenuate heat stress-induced autophagy-associated ultrastructural and transcriptional alterations in the testes.

### 3.5. Untargeted Metabolomic Profiling Reveals Alterations in Testicular Amino Acid and Arginine-Related Metabolism Under Heat Stress

**Figure 5:**
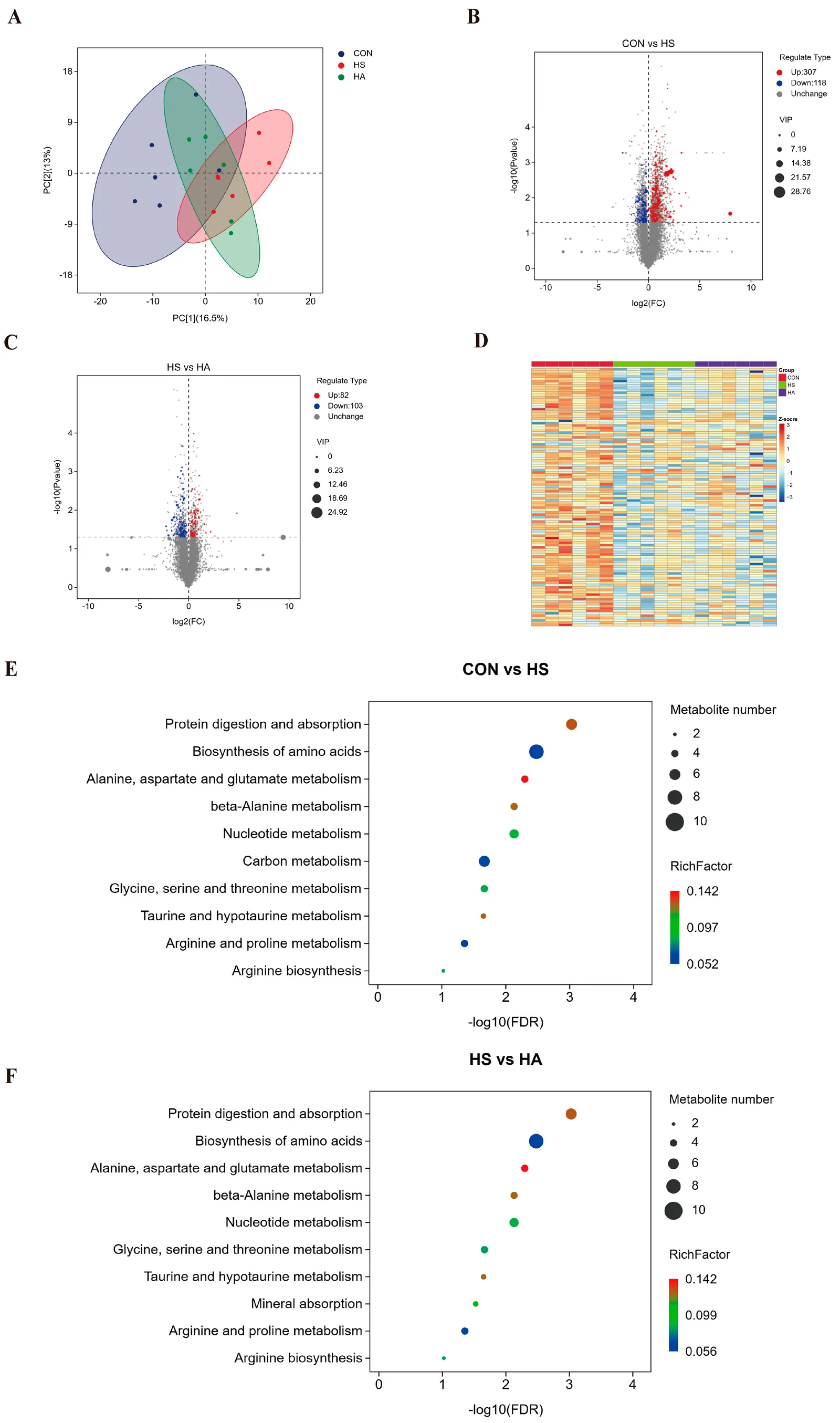
Untargeted metabolomic profiling of testicular tissues from Rongchang boars exposed to heat stress and dietary arginine supplementation. (**A**) Principal component analysis (PCA) score plot showing the distribution of testicular metabolomic profiles among the CON, HS, and HA groups. (**B**,**C**) Volcano plots showing the differential metabolites identified in the CON vs. HS comparison (**B**) and HS vs. HA comparison (**C**). Red and blue dots indicate significantly upregulated and downregulated metabolites, respectively, and gray dots indicate unchanged metabolites. Dot size represents the variable importance in projection (VIP) score. (**D**) Heatmap showing the relative abundance patterns of differential metabolites across the three groups. The color scale represents normalized metabolite abundance. (**E**,**F**) Kyoto Encyclopedia of Genes and Genomes (KEGG) pathway enrichment analysis of differential metabolites in the CON vs. HS comparison (**E**) and HS vs. HA comparison (**F**). Dot size represents metabolite number, and color indicates the rich factor. CON, thermoneutral control group fed the basal diet; HS, heat-stressed group fed the basal diet; HA, heat-stressed group fed the diet supplemented with 0.8% arginine; FC, fold change; FDR, false discovery rate.

Untargeted metabolomic profiling was performed to characterize testicular metabolic changes associated with heat stress and dietary arginine supplementation. First, principal component analysis (PCA) showed partial separation among the CON, HS, and HA groups, indicating treatment-associated shifts in testicular metabolism (Figure 5A). Consistently, differential metabolite analysis revealed substantial metabolic changes between groups. In the CON vs. HS comparison, 307 metabolites were significantly increased and 118 were significantly decreased (Figure 5B). In the HS vs. HA comparison, 82 metabolites were significantly increased and 103 were significantly decreased (Figure 5C). Furthermore, heatmap analysis showed distinct clustering patterns of differential metabolites among the three groups, further supporting heat stress-induced remodeling of the testicular metabolic profile (Figure 5D). To explore the functional relevance of these metabolic changes, KEGG pathway enrichment analysis was performed. Differential metabolites from both comparisons were mainly enriched in amino acid-related pathways, including biosynthesis of amino acids, arginine biosynthesis, and arginine and proline metabolism (Figure 5E,F). Metabolomic analysis indicated that heat stress disrupted testicular metabolic homeostasis, whereas dietary arginine supplementation reshaped amino acid- and arginine-related metabolic profiles under heat-stress conditions.

### 3.6. Dietary Arginine Supplementation Improves Circulating Arginine Availability and Testicular Arginine-Related Metabolism Under Heat Stress

**Figure 6:**
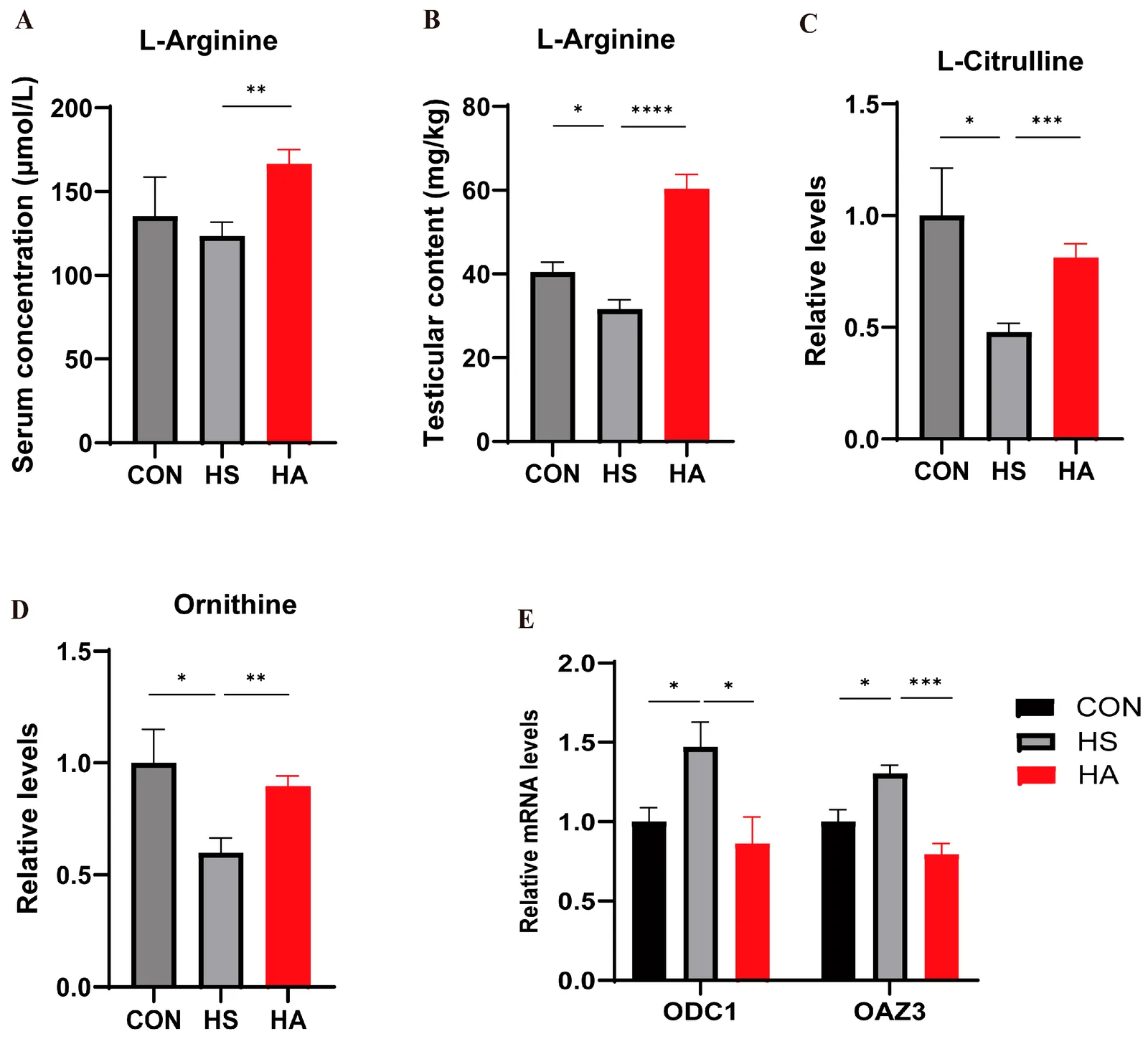
Effects of dietary arginine supplementation on circulating arginine availability and testicular arginine-related amino acid and polyamine metabolism in heat-stressed Rongchang boars. (**A**) Serum L-arginine concentration. (**B**) Testicular L-arginine content. (**C**) Relative abundance of testicular L-citrulline. (**D**) Relative abundance of testicular ornithine. (**E**) Relative mRNA expression levels of ornithine decarboxylase/polyamine pathway-associated genes, including *ODC1* and *OAZ3*, in the testes. CON, thermoneutral control group fed the basal diet; HS, heat-stressed group fed the basal diet; HA, heat-stressed group fed the diet supplemented with 0.8% arginine. Data are presented as mean ± SEM. Statistical analyses were performed using one-way ANOVA followed by LSD multiple-comparison tests. Asterisks indicate significant differences between the indicated groups: * *p* < 0.05; ** *p* < 0.01; *** *p* < 0.001; **** *p* < 0.0001.

Guided by the enrichment of amino acid- and arginine-related pathways in the untargeted metabolomic analysis, circulating and testicular indices of arginine metabolism were further examined. Accordingly, serum L-arginine concentration was significantly higher in the HA group than in the HS group (*p* < 0.05; Figure 6A), indicating enhanced circulating arginine availability after dietary arginine supplementation under heat-stress conditions. In the testes, L-arginine content was significantly reduced in the HS group compared with the CON group. However, this reduction was markedly alleviated in the HA group (*p* < 0.05; Figure 6B). Consistently, the relative abundances of L-citrulline and ornithine showed a similar pattern, with decreases under heat stress and increases after arginine supplementation (*p* < 0.05; Figure 6C,D). To further assess arginine-related polyamine regulation, qRT-PCR analysis showed that *ODC1* and *OAZ3* were significantly upregulated in the HS group compared with the CON group, but were reduced in the HA group compared with the HS group (*p* < 0.05; Figure 6E). Overall, these findings suggest that dietary arginine supplementation enhances circulating arginine availability and modulates heat stress-disrupted testicular arginine-related amino acid and polyamine metabolism in Rongchang boars.

## 4. Discussion

Heat stress is a major environmental factor that impairs growth performance, metabolic homeostasis, and male reproductive function in livestock and other mammals [25,26,27]. In the present study, cyclic heat-stress exposure induced clear physiological responses to thermal challenge in Rongchang boars, as evidenced by increased THI, respiratory rate, and rectal temperature. Consistently, heat-stressed animals exhibited reduced feed intake and body weight compared with thermoneutral controls, which agrees with the typical adaptive responses of pigs exposed to high ambient temperatures [26,28]. Under thermal challenge, pigs reduce feed intake to decrease metabolic heat production and increase respiratory activity to promote heat dissipation. Accordingly, the elevated respiratory rate and rectal temperature observed in the HS and HA groups indicated that both heat-stressed groups experienced marked thermoregulatory stress. At the molecular level, increased *HSF5* expression in the HS group further suggested heat-responsive transcriptional changes in the testes. Notably, dietary arginine supplementation did not prevent the development of heat stress-related physiological responses, indicating that its beneficial effects were unlikely to be mediated through direct reduction in heat load. Instead, arginine supplementation may alleviate heat stress-induced reproductive dysfunction through coordinated changes in metabolic and cellular stress-associated responses.

The reproductive consequences of heat stress were further reflected by alterations in epididymal sperm quality and testicular histological characteristics. In the present study, heat stress significantly reduced sperm viability, sperm concentration, and sperm motion parameters, including VCL, VSL, and VAP, indicating impaired sperm functional quality under thermal challenge [11,29,30,31]. These findings are consistent with the high sensitivity of developing germ cells, epididymal sperm function, and the testicular microenvironment to elevated temperatures [32]. The seminiferous epithelium provides a specialized niche that supports germ-cell development, meiotic progression, and spermiogenesis; therefore, structural alterations within this compartment may affect the maintenance of normal testicular function and sperm quality [10]. Consistent with this concept, heat stress induced epithelial disorganization, enlarged luminal spaces, increased vacuolated areas, and reduced seminiferous epithelial height, suggesting alterations in seminiferous tubule integrity. Although dietary arginine supplementation significantly improved sperm viability and VSL under heat-stress conditions, quantitative analysis showed no significant differences in vacuolated area or seminiferous epithelial height between the HS and HA groups. Therefore, the beneficial effects of arginine supplementation were more apparent in epididymal sperm functional parameters, whereas improvements in testicular histological characteristics appeared limited and should be considered as partial structural improvement rather than complete recovery.

Single-cell transcriptomic analysis further provided insights into the cellular changes associated with heat stress-induced testicular alterations. Normal testicular function depends on the coordinated maintenance of germ-cell populations, including spermatogonia, spermatocytes, and spermatids, together with the supportive functions of somatic cells within the testicular microenvironment [33,34,35,36]. In the present study, heat stress altered testicular cellular composition, as reflected by changes in the relative proportions of spermatocytes and spermatogonia. These changes may reflect differential sensitivity of specific germ-cell populations to thermal stress and alterations in cellular distribution rather than complete disruption of the spermatogenic process. Spermatocytes undergo active meiotic events and are considered particularly vulnerable to environmental stress; therefore, their altered proportion may be associated with the reduced sperm quality observed in heat-stressed animals. In addition to germ cells, Sertoli cells identified by scRNA-seq may represent an important component involved in the response to thermal stress, as they regulate germ-cell development, maintain the blood–testis barrier, and support testicular microenvironmental homeostasis [37,38]. Alterations in Sertoli cell-associated responses may therefore contribute to changes in testicular cellular homeostasis under heat stress conditions. The increased relative abundance of spermatogonia, particularly in the HA group, may reflect differences in germ-cell population distribution following heat exposure with or without arginine supplementation. Nevertheless, scRNA-seq-derived cell proportions should be interpreted cautiously because technical factors, including tissue dissociation efficiency and cell survival, may influence the apparent abundance of specific cell populations [36]. Given the relatively short duration of heat exposure, the epididymal sperm analyzed at the end of the experiment were likely already at advanced stages of development or undergoing epididymal transit when heat stress was initiated. Therefore, the observed reproductive changes should primarily be interpreted as early responses involving existing germ-cell populations, the testicular microenvironment, and epididymal sperm function rather than as alterations encompassing the entire spermatogenic process.

The KEGG enrichment results from scRNA-seq highlighted autophagy-related pathways, including mitophagy, suggesting that autophagy-associated responses may be involved in cellular adaptation to heat stress [36,37,38,39]. Autophagy is a conserved lysosome-dependent process that contributes to cellular homeostasis by removing damaged proteins and organelles during stress conditions. However, dysregulated autophagy responses under excessive stress may be associated with cellular dysfunction and tissue injury. Consistent with this concept, TEM observations in the present study revealed ER swelling, ultrastructural disorganization, and autophagy-associated ultrastructural alterations in testicular cells following heat exposure. These structural changes were accompanied by increased expression of autophagy-associated genes (*GABARAPL1*, *ULK1*, and *BECN1*) and the cellular stress-related gene *CALM3*, suggesting altered autophagy-associated and ER-associated stress responses [38]. Although *CALM3* is not a canonical autophagy marker, calmodulin-dependent calcium signaling has been implicated in cellular stress responses and may influence autophagy-related processes under stress conditions [40]. Therefore, *CALM3* was considered as a stress-associated indicator rather than a direct marker of autophagic activity in the present study. *ULK1* and *BECN1* are involved in autophagy initiation, whereas *GABARAPL1* contributes to autophagosome formation and maturation. Dietary arginine supplementation reduced autophagy-associated ultrastructural alterations and decreased *ULK1* and *BECN1* expression compared with the HS group, suggesting that arginine supplementation may alleviate heat stress-associated cellular stress responses involving autophagy-related processes. Nevertheless, because autophagic flux and protein-level markers were not evaluated in the present study, these findings should be interpreted as changes in autophagy-associated responses rather than direct evidence of altered autophagic activity.

Metabolic remodeling represents another important feature associated with heat stress-induced testicular dysfunction [41,42,43]. Consistent with this concept, untargeted metabolomic profiling revealed treatment-associated alterations in testicular metabolic profiles. Differential metabolites were mainly enriched in amino acid-related pathways, including amino acid biosynthesis, arginine biosynthesis, and arginine and proline metabolism. These pathways may contribute to energy metabolism, redox regulation, cellular proliferation, and stress adaptation [44]. Among these metabolic processes, arginine metabolism is particularly relevant because arginine is a conditionally essential amino acid during physiological stress and serves as a precursor for nitric oxide, ornithine, and polyamines, which participate in vascular regulation, antioxidant defense, cellular growth, and reproductive function [12,16]. In the present study, dietary arginine supplementation increased serum L-arginine concentrations under heat-stress conditions, indicating improved circulating arginine availability. In contrast, heat stress reduced testicular L-arginine content and decreased the relative abundances of L-citrulline and ornithine. The coordinated reduction of these arginine-related metabolites suggests a broad disturbance of testicular arginine metabolic homeostasis rather than a simple increase in downstream arginine utilization. Dietary arginine supplementation partially alleviated these metabolic alterations, suggesting that arginine may contribute to the restoration of arginine-related metabolic balance under heat-stress conditions. The altered expression patterns of *ODC1* and *OAZ3* further suggest changes in the ornithine decarboxylase/polyamine regulatory pathway [45]. *ODC1* encodes ornithine decarboxylase, a rate-limiting enzyme involved in the conversion of ornithine to putrescine during polyamine biosynthesis. Meanwhile, *OAZ3*, a member of the ornithine decarboxylase antizyme family, participates in the regulation of ODC activity and has been associated with male reproductive development [46]. In the present study, *ODC1* and *OAZ3* expression was highest in the HS group and was reduced following dietary arginine supplementation. However, this expression pattern does not necessarily indicate enhanced polyamine biosynthesis under heat stress. Instead, increased *ODC1* and *OAZ3* expression may represent a compensatory or feedback response to disturbed arginine and ornithine metabolism. Consistently, the reduction in ODC1 and *OAZ3* expression in the HA group, together with partially restored L-arginine, L-citrulline, and ornithine levels, suggests that dietary arginine supplementation may alleviate heat stress-associated alterations in the testicular ornithine decarboxylase/polyamine regulatory axis. Therefore, arginine supplementation appears to modulate, rather than simply stimulate, testicular arginine-related amino acid and polyamine metabolism under heat-stress conditions.

Together, these findings suggest that heat stress-induced reproductive alterations are associated with coordinated changes in germ-cell composition, arginine-related metabolism, and autophagy-associated cellular homeostasis [47]. Dietary arginine supplementation increased circulating arginine availability and partially improved testicular arginine-related metabolic homeostasis, which may contribute to cellular stress adaptation under thermal challenge. In parallel, arginine supplementation was associated with reduced ER swelling, altered autophagy-associated ultrastructural changes, and changes in the expression of autophagy-associated and stress-related genes. These metabolic and cellular responses may collectively contribute to improved sperm viability and VSL, as well as partial improvement of testicular histological characteristics in heat-stressed Rongchang boars. Overall, these findings suggest that arginine-related metabolic modulation represents a potential nutritional strategy for supporting male reproductive function during thermal stress.

Several limitations should be acknowledged. First, although transcriptomic analysis, ultrastructural observation, and qRT-PCR results indicated alterations in autophagy-associated responses, autophagic flux and protein-level markers were not directly evaluated. Therefore, these findings should be interpreted as changes associated with autophagy-related cellular responses rather than definitive evidence of altered autophagic activity. Because the TEM analysis was performed on testicular tissue sections without cell-type-specific labeling, the ultrastructural alterations observed in the present study could not be reliably assigned to Sertoli cells, Leydig cells, or specific germ-cell populations. Therefore, these findings are conservatively described as alterations in testicular cells. Second, the heat-stress exposure period was limited to 9 days, representing an early thermal challenge rather than a complete spermatogenic cycle in boars. Third, heat stress-induced reductions in feed intake may have resulted in differences in actual arginine intake among treatments. Therefore, the beneficial effects observed following arginine supplementation should be interpreted considering both dietary arginine supplementation and heat stress-induced changes in nutrient intake. Finally, additional mechanistic studies are required to establish causal relationships among arginine metabolism, cellular stress responses, and reproductive outcomes.

## 5. Conclusions

In conclusion, cyclic heat stress compromised epididymal sperm quality and testicular histological characteristics in Rongchang boars. These alterations were accompanied by changes in germ-cell composition, arginine-related metabolic homeostasis, and autophagy-associated cellular responses in the testes. Dietary arginine supplementation improved sperm viability and straight-line velocity, partially improved testicular histological characteristics, increased circulating arginine availability, and reshaped testicular arginine-related amino acid and polyamine metabolic profiles. Furthermore, these beneficial effects were associated with reduced endoplasmic reticulum swelling, altered autophagy-associated ultrastructural changes, and changes in the expression of autophagy-associated and stress-related genes under heat-stress conditions. Overall, dietary arginine supplementation alleviated heat stress-induced testicular dysfunction, which was associated with coordinated changes in arginine-related metabolism and autophagy-associated cellular responses, supporting its potential application as a nutritional strategy to maintain male reproductive function during thermal stress.

## Figures and Tables

**Table 1:** Ingredient composition of the experimental diets (%, as-fed basis).

Ingredient	6–15 kg BW		15–30 kg BW	
	Control Diet	0.8% L-Arginine Diet	Control Diet	0.8% L-Arginine Diet
Corn	26.24	27.21	44.41	45.38
Extruded corn	35.34	35.34	17.67	17.67
Dehulled soybean meal	7.95	7.83	7.95	7.83
Extruded full-fat soybean	7.90	7.90	7.90	7.90
Whey powder	7.04	7.04	7.04	7.04
Soy protein concentrate	3.54	3.54	3.54	3.54
Sucrose	2.46	2.46	2.46	2.46
Fish meal, 67% CP	2.90	2.90	2.90	2.90
Soybean oil	1.59	1.59	1.59	1.59
L-lysine hydrochloride	0.37	0.37	0.37	0.37
DL-methionine	0.06	0.06	0.06	0.06
L-threonine	0.09	0.09	0.09	0.09
L-tryptophan	0.02	0.02	0.02	0.02
L-alanine	1.70	0.00	1.70	0.00
L-arginine	0.00	0.82	0.00	0.82
Choline chloride, 50%	0.15	0.15	0.15	0.15
Calcium carbonate	0.64	0.64	0.64	0.64
Dicalcium phosphate dihydrate	0.77	0.80	0.77	0.80
Feed-grade sodium chloride	0.20	0.20	0.20	0.20
Vitamin premix	0.05	0.05	0.05	0.05
Benzoic acid	0.30	0.30	0.00	0.00
Zinc oxide	0.20	0.20	0.00	0.00
Mineral premix	0.50	0.50	0.50	0.50
Total	100.00	100.00	100.00	100.00

Note: BW, body weight; CP, crude protein. The experimental diets were formulated according to the nutrient requirements for swine in China. Values are expressed as percentages of the diet on an as-fed basis unless otherwise indicated. The 0.8% L-arginine diets were formulated by adjusting ingredient proportions. The premix provided the following per kg of complete diet: Cu, 20.00 mg; Fe, 100.00 mg; Zn, 100.00 mg; Mn, 20.00 mg; Se, 0.30 mg; I, 0.15 mg; vitamin A, 12,000 IU; vitamin D3, 2400 IU; vitamin E, 100 IU; vitamin K3, 4.80 mg; biotin, 0.48 mg; folic acid, 4.00 mg; available niacin, 40.00 mg; pantothenic acid, 25.00 mg; riboflavin (vitamin B2), 7.20 mg; thiamine (vitamin B1), 2.00 mg; vitamin B6, 3.60 mg; and vitamin B12, 0.025 mg.

**Table 2:** Primer sequences used for qRT-PCR.

Gene	Primer Sequence (5′–3′)	GenBank Accession Number
*HSF5*	F: CTGGATCTCCTGCTATACTCAC R: CAGACCCTACTGGCTAATTCAC	XM_003131648.3
*GABARAPL1*	F: CTGATCTGGACAAGAGGAAGTACC R: GGCCACGTACAGAAAGTAGTCT	NM_001190287.1
*ULK1*	F: GGGGAAGGAAATCAAGATCCTC R: CTGCAATCTGCTGTAGGAAGAG	XM_021072523.1
*P62*	F: TTTCCTCAAGAACGTAGGGGAG R: CTTCTCTTCCCTCCATGTTCCA	XM_003123639.4
*BECN1*	F: GATGGTGGCTTTCCTGGACTGTG R: ACTGCCTCCTGTGTCTTCAATCTTG	NM_001044530.1
*CALM3*	F: AGCTGACTGAGGAGCAGATTG R: AACTCCTTGGTGGTGATAGTGC	NM_001244209.1
*DAPK3*	F: GTGGAAGACCACTATGAGATGGG R: CTTGATGAACTTGGCCGCATAC	XM_021084109.1
*ATP2A2*	F: GGCTGATGGTGCTGAAAATC R: GGCTGGTAGATGTGTTGCTA	XM_021072075.1
*m* *TOR*	F: CTAGAAATGAGGAAGTGGGTGG R: CAATGTGGGGTACTTCCTGTAG	XM_003127584.6
*ODC1*	F: TTTGGAGCGGGCGAGGGATC R: CGAAGACACAGCGGGCATCAG	NM_001122983.1
*OAZ3*	F: AGCAGCAAGTGGTGGATTTG R: CTTAGTGACCAGAGACTCTTCC	NM_001122996.2
*β-actin*	F: GACCTCAAGTACCCCATCGA R: TTGTAGAAGGTGTGGTGCCAGAT	ON164673.1

Note: F, forward primer; R, reverse primer; qRT-PCR, quantitative real-time PCR.

## Data Availability

The data supporting the findings of this study are available from the corresponding author upon reasonable request.
